# Canine vector-borne infections of working dogs of the Sri Lanka Air Force, and free roaming, and privately owned dogs

**DOI:** 10.1038/s41598-024-71148-1

**Published:** 2024-10-07

**Authors:** P. S. Jayatilaka, R. A. S. Ranatunga, H. S. U. Wijerathna, A. D. S. Fernando, K. M. H. Jinarathne, N. G. R. K. Naullage, S. N. S. Silva, K. Thananjayan, L. K. H. R. T. Amarasiri, N. P. K. Jayasundara, M. C. K. Mallawa, A. Dangolla, S. S. Iddamaldeniya, S. M. N. S. Samarakoon, A. G. M. L. K. Dayananda, A. M. M. Nazeem, R. S. Rajakaruna

**Affiliations:** 1 Sri Lanka Air Force, Katunayake, Sri Lanka; 2 Postgraduate Institute of Science, University of Peradeniya, Peradeniya, Sri Lanka; 3 Department of Molecular Biology and Biotechnology, Faculty of Science, University of Peradeniya, Peradeniya, Sri Lanka; 4 Suwana’ Pet Care Animal Hospital, Nagoda, Kalutara, Sri Lanka; 5 Government Veterinary Hospital, Wennappuwa, Sri Lanka; 6 Department of Veterinary Clinical Sciences, Faculty of Veterinary Medicine and Animal Science, University of Peradeniya, Peradeniya, Sri Lanka; 7 Veterinary Research Institute, Gatambe, Peradeniya, Sri Lanka; 8 SN308 The Gardens, OIST, Kunigami District, Onna Village, 1919-1 Tancha, Okinawa, Japan; 9 Department of Zoology, Faculty of Science, University of Peradeniya, Peradeniya, Sri Lanka

**Keywords:** Dogs, Asymptomatic cases, Vector-borne diseases, Military working dogs, Genetics, Microbiology, Diseases

## Abstract

Canine vector-borne infections (CVBIs) are a global health problem. The military working dogs of Sri Lanka die at an early age, and CVBIs have been a leading speculated cause. We examined CVBIs in the working dogs of the Sri Lanka air force (SLAF) and free-roaming dogs (FRDs) and privately owned dogs (PODs) country-wide. Giemsa-stained smears were prepared and conventional PCR-positive DNA was subjected to sequencing and phylogeny. Of the 668 dogs sampled, 212 (31.7%) had one or more CVBIs. The prevalence of infections among the FRDs (40.0%) was significantly higher than SLAF working dogs (30.0%; χ^2^ = 10.5216; *p* = 0.0012) and PODs (26.2%; χ^2^ = 5.3414, *p* = 0.0208) but not between SLAF dogs and PODs (χ^2^ = 1.7655, *p* = 0.1838). Many infected dogs were asymptomatic (57.4%), which was higher among the FRDs. Seven infectious agents were identified: *Babesia gibsoni*, *B. canis, Ehrlichia canis*, *Anaplasma platys*, *Leishmania* sp., *Hepatozoon canis*, and filaria worms. The most common infection was *B. gibsoni* (13.8%), followed by *E. canis* (9.9%). Three tick species: *Rhipicephalus linneai*, *Rhipicephalus haemaphysaloides* and *Haemophysalis bispinosa* were found infesting the dogs. The SLAF dogs were thoroughly quarantined upon arrival, but the infection prevalence was similar to PODs.

## Introduction

Canine vector-borne infections (CVBIs) represent many infectious diseases of major significance for canine health globally, especially in tropical and subtropical countries. The etiology of CVBIs involves pathogens like protozoans, helminths, bacteria, and viruses, including *Anaplasma, Babesia, Ehrlichia, Hepatozoon, Dirofilaria, Trypanosoma, Borrelia, Leishmania, Mycoplasma,* and *Rickettsia.* These pathogens are transmitted through arthropod vectors like ticks, lice, fleas, mosquitoes, sandflies, tabanid flies, and triatomines. Clinical manifestation of CVBIs can vary from asymptomatic cases to severe health implications depending on the pathogenicity of the causative agent and the presence of co-infections complicating the diagnosis, host immunity, and environmental factors^1–4^.

Many CVBIs have been reported in dogs in Sri Lanka, including *Babesia gibsoni, Babesia canis*^5–7^, *Anaplasma platy*^8^, *Ehrlichia canis*^6,8, 9^*, Hepatozoon canis*^6,10^, filaria worms belonging to *Brugia malayi*^11^, *Dirofilaria repens*^11–13^ and *Trypanasoma evansi*^14^. The occurrence of *Leishmania* in dogs in Sri Lanka is suspected as amastigotes from two out of 151 dogs observed but not confirmed^15,16^. *Haemotropic mycoplasmas* (formerly known as *Haemobartonella canis*) has also been reported in 1973 from Sri Lanka^17^. Canine rickettsial infections were first reported in 1962^18^ and later serological evidence of exposure of dogs to rickettsial infections identified *Rickettsia conorii, Rickettsia typhi, and Orientia tsutsugamushi*^19^. Co-infection of several species of *Anaplasma, Babesia, Ehrlichia, Rickettsia,* and *Hepatozoon* is common among the dogs brought to veterinary care facilities^20^. However, detailed data such as geographic distribution of the disease, epidemiology, and vectors of CVBIs are not available, scant, or outdated. Consequently, developing new and endemic foci of CVBIs in non-endemic areas could occur without efficient veterinary and public health surveillance networks, possibly resulting in a rapid spread of the infection among the dog populations.

Dogs infected with CVBIs may show varying clinical presentation, pathogenicity, and response to therapy^21^. As early as 1953, piroplasmosis in dogs in Sri Lanka was studied, describing *B. gibsoni,* the small canine *Babesia* infections in dogs^22^, its treatment^23^, and pathology^24^. Clinical determinants of the infection include virulence of the agent, age, gender, and the dog's immune status^25,26^. Clinical presentation and pathogenicity of the disease show variation among the pedigreed and stray dogs^27^. Young adults of dog breeds like German Shepherds, Dobermann, and Pomeranians show the highest susceptibility to babesiosis and ehrlichiosis, while Rottweilers, Labradors, Boxers, and crossbred dogs show susceptibility at any age group^20^. However, non-pedigree, mixed breeds and stray dogs show more resistance and tolerance to these infections, probably due to diverse ancestry and higher genetic diversity^20^. Most of these CVBIs represent a substantial diagnostic challenge for veterinarians because clinical signs are often absent, diffuse, and overlapping; co-infections with two or more pathogens further this problem^28^.

CVBIs are a significant health concern among military working dogs^29^. There are about 500 military working dogs in the Sri Lanka Army, Navy, Air Force, and the Police. Most of these dogs are imported from various countries, mainly from the UK, Germany, and the Netherlands, and some are locally bred (Personal communication with the veterinarians in the military and Police). The working dogs die early, and the average age at death is 6.1 years for those in the Sri Lanka Police^30^. Moreover, 75% of the dogs belonging to the Sri Lanka Air Force (SLAF) and Army have died, presumably due to CVBIs; although suspected, these have not been confirmed or properly diagnosed (personal communication with the Squadron Leader and the Manager of Animal Husbandry Project at the SLAF, Katunayake). There is a proper quarantine and screening process for all the dogs before introducing them to the military unit; they acquire the diseases through the arthropod vectors once they have been in the kennels of the military or Police. There is an urgent need to provide baseline data on the types of infections and their prevalence in dogs; therefore, the study investigated the CVBIs in the military working dogs in the SLAF and free-roaming dogs (FRDs) and privately owned dogs (PODs).

## Results

### Study animals

**Fig. 1:**
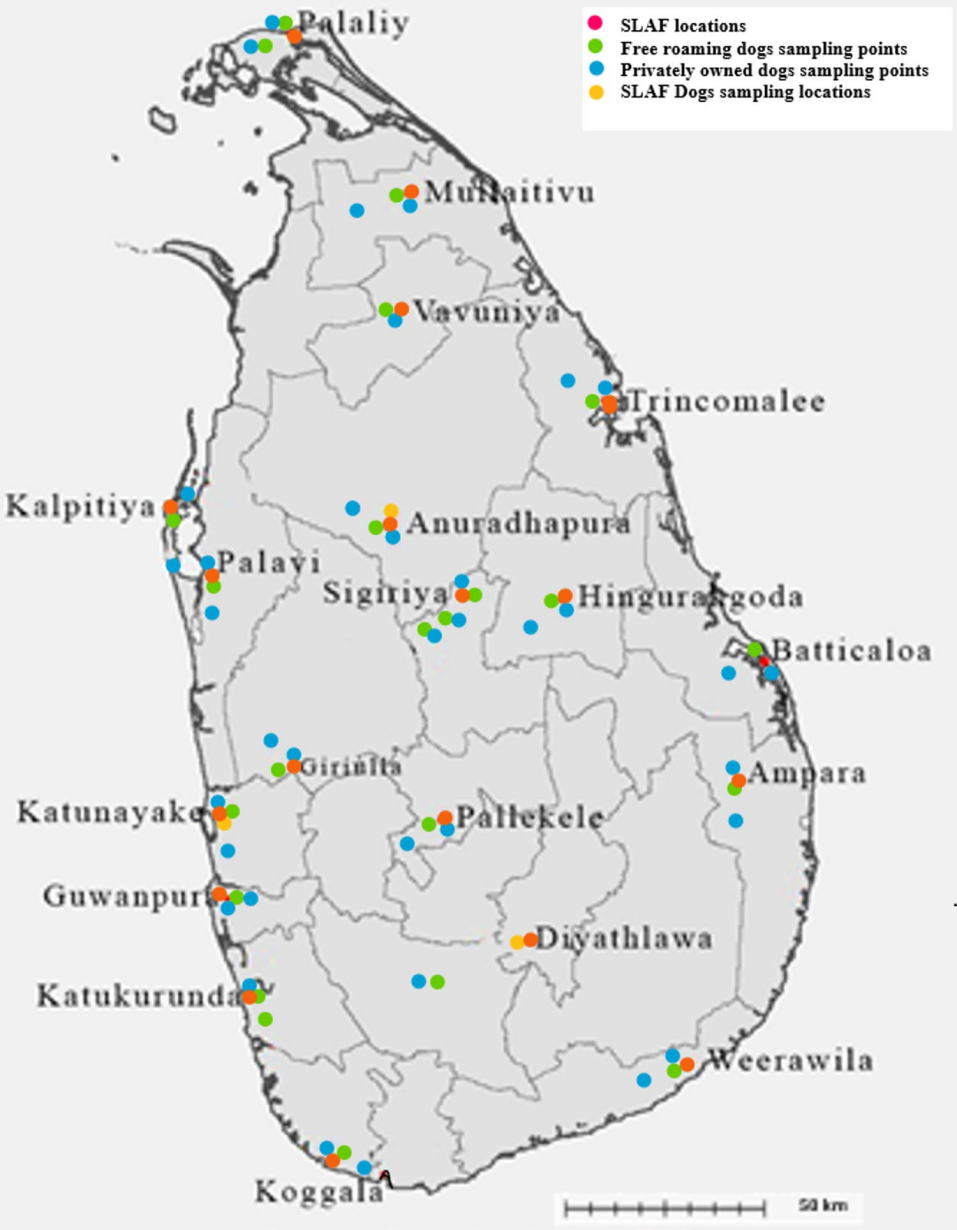
Map of Sri Lanka showing the sampling sites.

Blood samples and background information of dogs were collected from 18 districts (out of 25) in Sri Lanka (Fig. 1). A total of 668 dogs were sampled, comprising 173 from the three SLAF establishments, 205 FRDs (115 healthy dogs living close to the SLAF establishments and 90 from veterinary clinics island-wide), and 290 PODs (90 healthy dogs living close to the SLAF and 200 from veterinary clinics island-wide; Table 1). All the SLAF dogs were purebred, the FRDs were all mongrels, and the PODs were a mixture of purebred, mongrel, or mixed breeds. Military working dogs at the SLAF belonged to seven breeds. They were either imported or the parents were imported, but some were locally bred. There were 271 (40.8%) male dogs and 397 (59.4%) female dogs, comprising 560 (83.8%) adults and 108 (16.2%) puppies.

**Table 1:** Prevalence of single and mixed infections among the Sri Lanka Air Force military working dogs, free-roaming and owned dogs from 18 districts in Sri Lanka (Blood smear positive data; n = 668).

Parasite species	Prevalence n (%)			
	SLAF* (n = 173)	Free-roaming (n = 205)	Privately owned (n = 290)	Overall (n = 668)
Single infections				
*Babesia gibsoni*	15 (8.7)	26 (12.7)	40 (13.8)	81 (12.1)
*Babesia canis*	0.0	0.0	1 (0.3)	1 (0.1)
*Ehrlichia canis*	13 (7.5)	13 (6.3)	19 (6.5)	45 (6.7)
*Anaplasma platys*	8 (4.6)	2 (0.9)	4 (1.3)	14 (2.1)
*Hepatozoon canis*	0.0	0.0	1 (0.3)	1 (0.1)
*Leishmania* sp.	0.0	2 (0.9)	0.0	2 (0.2)
Microfilaria	0.0	0.0	0.0	0.0
Total (Single)	36 (20.8%)	43 (21.0%)	65 (22.4%)	144 (21.6%)
Mixed infections				
*B. gibsoni* + *E. canis*	0.00	3 (1.4)	4 (1.3)	7 (1.0)
*B. gibsoni* + Microilariae	0.00	1 (0.4)	2 (0.6)	3 (0.4)
*B. gibsoni* + *A. platys*	0.00	1 (0.4)	0.00	1 (0.1)
*E. canis* + *A. platys*	2 (1.1)	6 (2.9)	5 (1.7)	13 (1.9)
*B. canis* + *A. platys* + Microfilariae	1 (0.5)	0.00	0.00	1 (0.1)
Total (Mixed)	3 (1.8%)	11 (5.4%)	11 (3.8%)	25 (3.7%)
Overall				
*Babesia gibsoni*	15 (8.7)	31 (15.1)	46 (15.7)	92 (13.8)
*Babesia canis*	–	–	1(0.3)	1(0.6)
*Ehrlichia canis*	15 (8.7)	22 (10.73)	28 (9.7)	66 (9.9)
*Anaplasma platys*	11 (6.4)	9 (4.4)	9 (0.3)	29 (4.3)
*Hepatozoon canis*	–	–	1 (0.3)	1 (0.2)
*Leishmania* sp.	–	2 (1)	–	2 (0.3)
Microfilariae	1 (0.6)	1 (0.5)	2 (0.7)	4 (0.6)
Grand total	42 (24.3%)	64 (31.2%)	85 (29.3%)	191 (28.6%)

*Sri Lanka air force.

### Prevalence and types of vector-borne infections

**Fig. 2:**
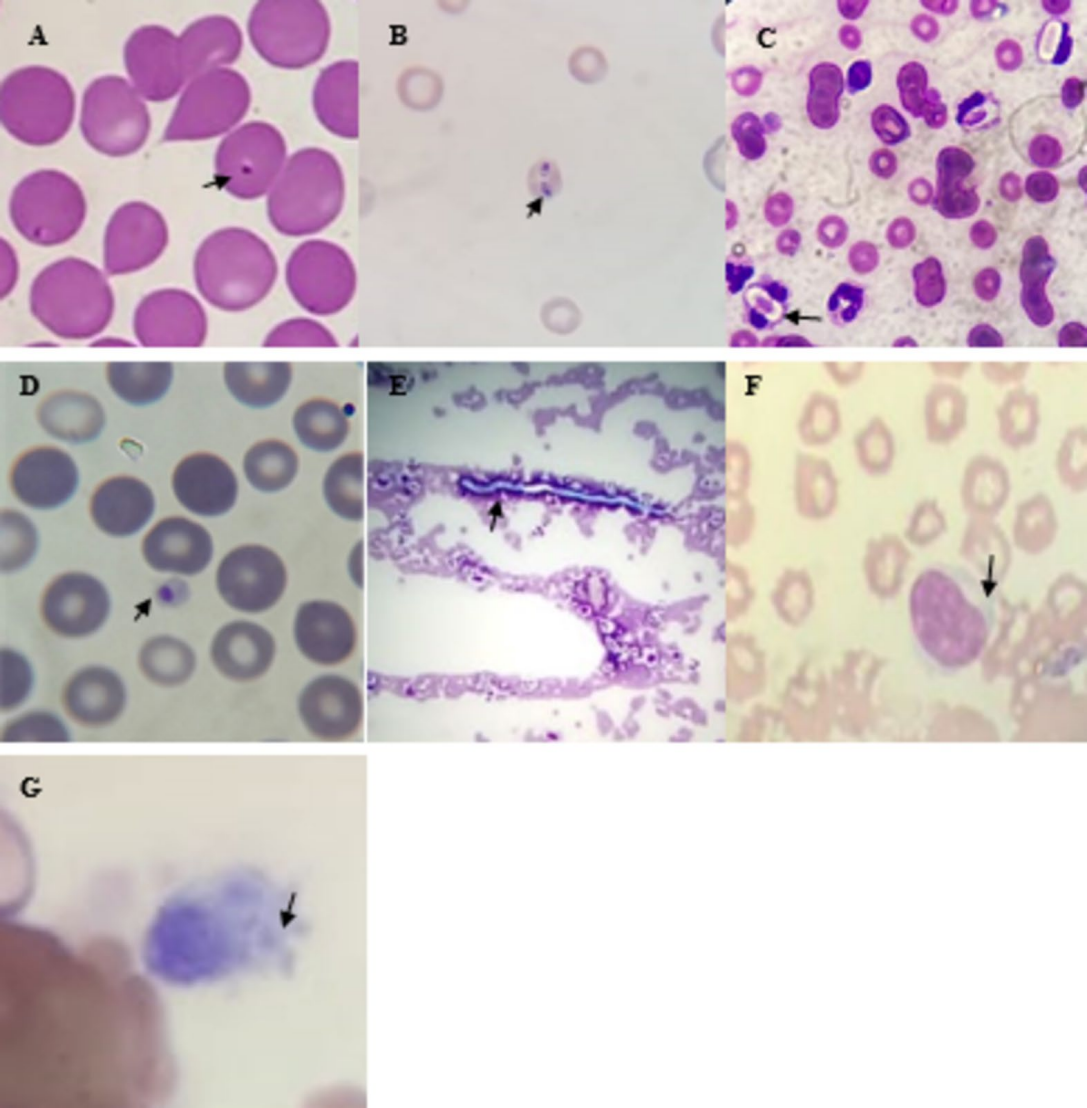
Giemsa-stained blood smears from infected dogs under light microscopy 100×. (**A**) *Babesia gibsoni* trophozoites in red blood cells; (**B**) *Babesia canis* pyriform-shaped trophozoites in red blood cells; (**C**) *Hepatozoon canis* intra-cytoplasmatic ellipsoidal-shaped gamonts in a neutrophil; (**D**) *Anaplasma platys* with the appearance of an inclusion body inside a platelet; (**E**) Microfilaria and (**F**) *Leishmania a*mastigotes

Based on microscopy and PCR results, overall, 212 dogs (31.7%) tested positive for one or more pathogens (Fig. 2). Of the infected dogs, 187 (88.2%) tested positive for a single infection, while 25 tested positive for more than one infection (11.8%).

The prevalence of infection in the dogs from the SLAF, FRDs, and PODs was 30.0%, 40.0% and 26.2%, respectively. The prevalence of infections among the FRDs was significantly higher than the SLAF working dogs (Chi-square test, χ^2^ = 10.5216; *p* = 0.0012) and PODs (χ^2^ = 5.3414, *p* = 0.0208) but not between SLAF dogs and PODs (χ^2^ = 1.7655, *p* = 0.1838). Among the infected dogs, many were asymptomatic (57.4%); this was significantly higher among FRDs (96.3%) compared to the SLAF (41.1%) and PODs (38.2%; *p* < 0.0001) but not between SLAF dogs and PODs (χ^2^ = 0.005, *p* > 0.9380). There was no sex predilection showing a difference in the prevalence of infection between male (9.6%) and female (15.7%; Table 2) dogs (Chi-square test, *p* > 0.05).

**Table 2:** Prevalence of infection with sex, age and presence of clinical signs among the different dog categories.

Host examined (n)	Prevalence n (%)			
	SLAF* (n = 173)	Free-roaming (n = 205)	Privately owned (n = 290)	Overall (n = 668)
Males (271)	10.9	7.8	10.0	9.6
Females (397)	11.6	18.5	16.2	15.7
Adults (560)	16.8	22.4	24.5	21.9
Puppies (108)	5.8	3.9	1.7	3.4
With clinical signs^1^	23/30 (76.7)	2/2 (100.0)	47/53 (88.7)	72/85 (84.7)
Without clinical signs^2^	16/143 (11.2%)	52/203 (25.6)	29/237 (12.2)	97/483 (20.1)
Total	39 (22.5%)	54 (26.3%)	76 (26.2%)	169 (25.3%)

*Sri Lanka Air Force; 1, Smears positive and with clinical signs; 2, Smears positive but without clinical signs.

Seven types of pathogens: *Babesia gibsoni, Babesia canis, Ehrlichia canis, Hepatozoon canis, Anaplasma platys Leishmaia* sp.*,* and filaria worms were recorded from microscopic examination of thin blood smears (Table 1; Fig. 2). Out of these, *B. gibsoni*, *B. canis*, *E. canis, H. canis*, and* A. platys* were confirmed by sequencing. Babesiosis was the most common disease among dogs island-wide (16.6%), caused by two parasites: *B. gibsoni* (16.3%) and *B. canis* (0.3%), contributing a significantly higher number by *B. gibsoni* (Fisher’s Exact test, χ^2^ = 80.8119, *p* < 0.0001). The second most common infection was *E. canis* (9.7%) followed by *A. platys* (4.3%). This distribution was similar in the three dogs categories. There was a difference in the prevalence of *B. gibsoni* in SLAF dogs (8.7%) and FRDs (23.4%; χ^2^ = 14.6851, *p* = 0.0001) and PODs (15.7%; χ^2^ = 4.8991, *p* = 0.0268) and also between FRDs and PODs (χ^2^ = 4.4532; *p* = 0.035). However, the prevalence of *A. platys* was higher in SLAF dogs (6.4%) compared to the other dogs (3.6%; χ^2^ = 7.274, *p* = 0.007). *Babesia canis* was found only in two dogs: one from a POD in Colombo (Sky Pet Hospital, Colombo) and another from the SLAF as a mixed infection with *A. platys* and filaria worms. The lowest prevalence of mixed infections was reported in the SLAF dogs (1.8%), and highest among the FRDs (5.4%) but the difference was not significant (Fisher's Exact test, χ^2^ = 0.0982, *p* > 0.05).

*Leishmania* sp. and *H. canis* occurred only as single infections. Other infections occurred as mixed infections of two or three parasites of various combinations (Table 1). The most common mixed infection was *E. canis* and *A. platys* (13 dogs) followed by *B. gibsoni* and *E. canis* (seven dogs). Even though mixed infections were rare among the SLAF dogs, one dog had *B. canis, A. platys* and filaria infections (Table 1). The least common single infection was *H. canis* reported from one POD in Borella, Colombo, while *Leishmania* was found only in FRDs in Katunayake. Filaria worms were always found as mixed infections (four dogs; Table 1). All the dog categories harboured *B. gibsoni, E. canis, A. platys* and filaria worms.

### DNA sequencing and phylogeny

#### *Babesia* species

**Fig. 3:**
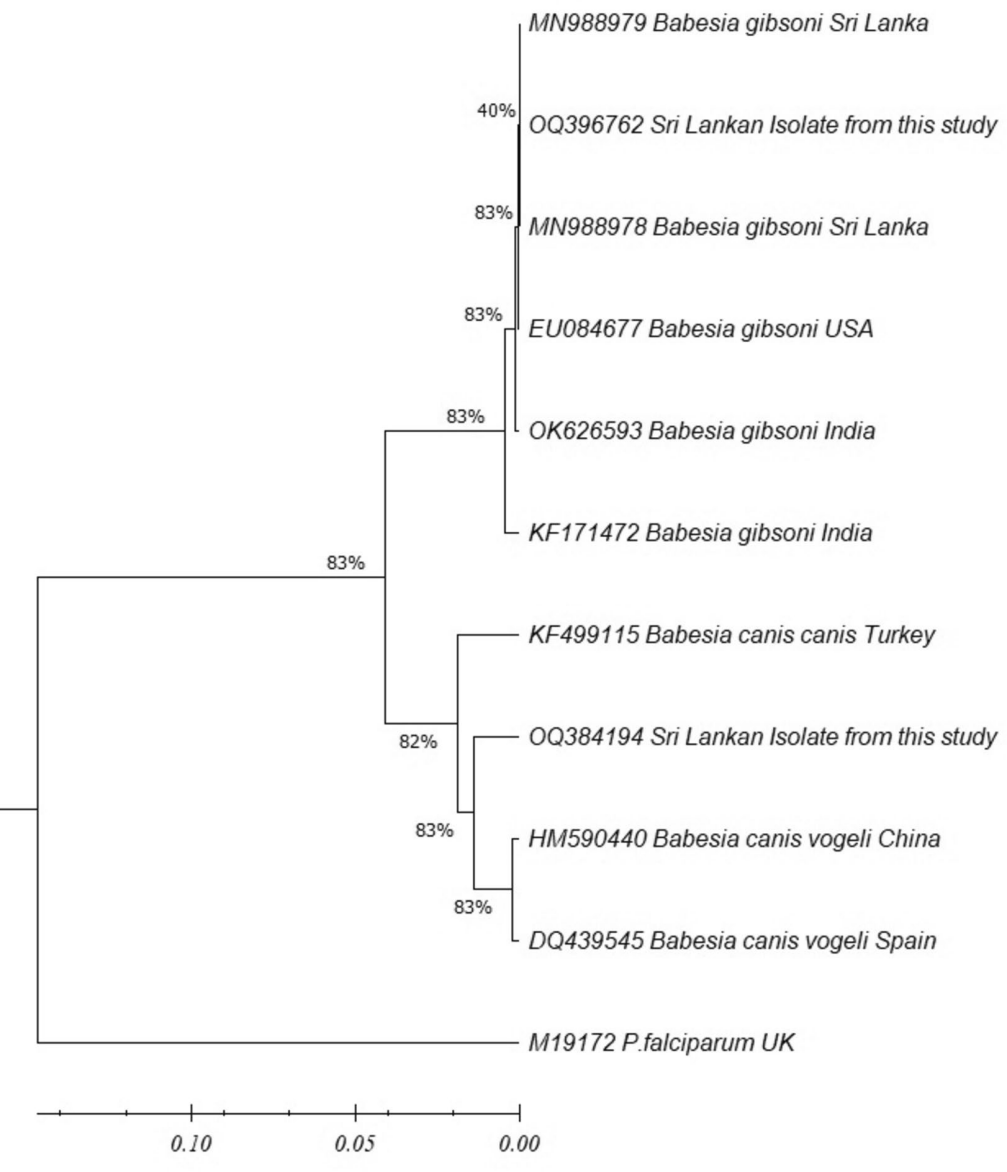
Phylogenetic tree of *Babesia* specie*s* isolates of this study based on the 18S ribosomal RNA (1*8S rRNA*) gene complete sequence with the neighbor-joining algorithm (GenBank accession numbers are indicated after species name)

The sequence for *B. gibsoni* was submitted to the GenBank database (accession number OQ396762). The phylogenetic analysis of the sequence confirmed the species as *B. gibsoni* (Fig. 3). The sequence was identical to the sequences isolated previously from Sri Lanka, India and the USA. The sequence of *B. canis* (GenBank accession number OQ 384194) in the phylogenetic tree shows its close affinities to *B. canis vogeli* isolates in Spain and China (Fig. 3). The tree was constructed using the neighbor-joining method, and the numbers above the internal nodes indicate the percentages of 1000 bootstrap replicates that supported the branch.

#### *Ehrlichia canis*

**Fig. 4:**
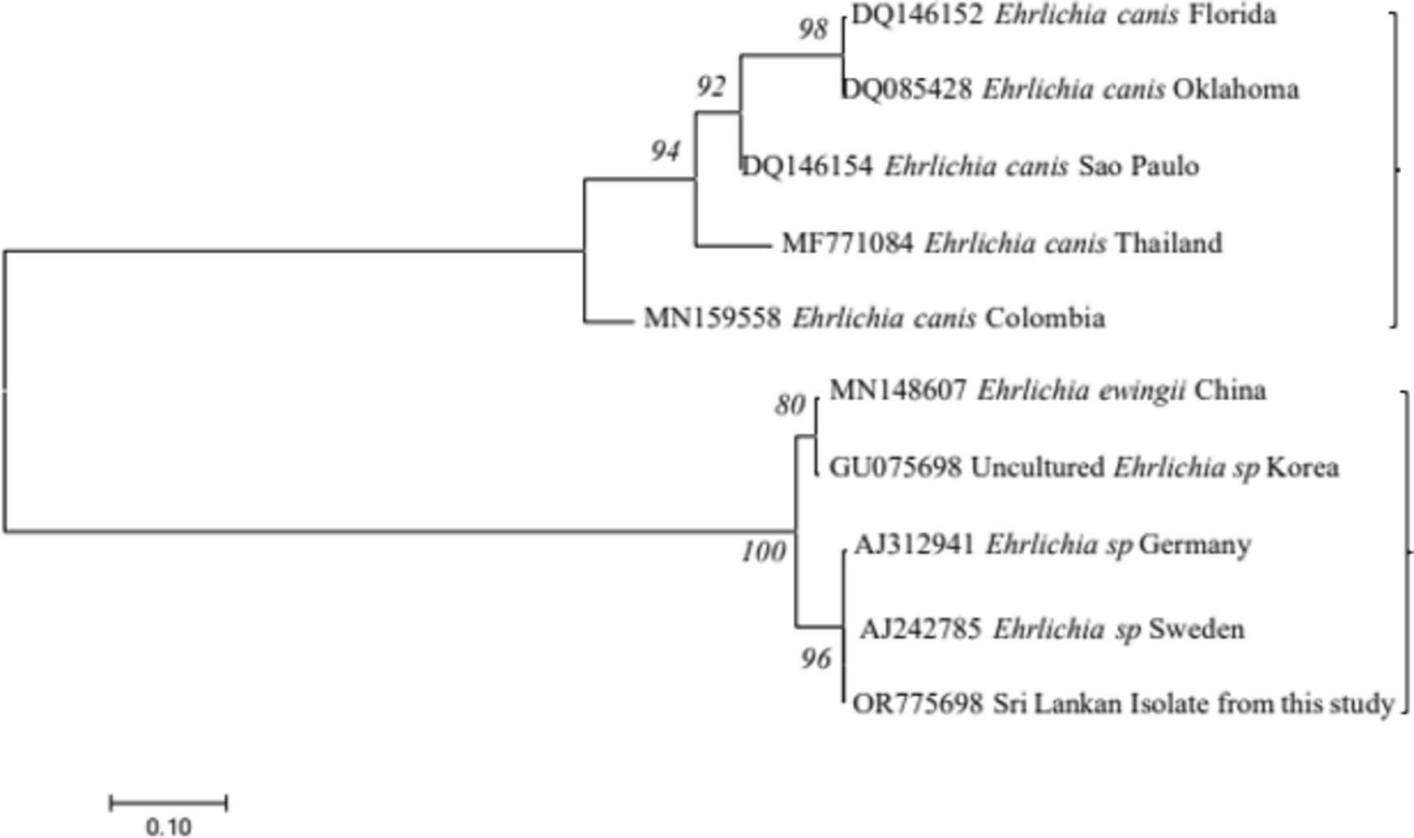
Phylogenetic tree of *Ehrlichia canis* based on the 16S ribosomal RNA partial sequence with the neighbor-joining algorithm (GenBank accession numbers are indicated after species name)

The phylogenetic analysis of the sequence confirmed the species as *Ehrlichia* (GebBank Accession No. OR775698)*.* The phylogenetic analysis (Fig. 4) revealed that the species identified from this study have close affinities to the *Ehrlichia sp.* isolates in Sweden (AJ242785). But was independent of *E. canis* reported in Thailand (MF 771084), USA (DQ146152, DQ085428) and Brazil (DQ146154). The tree was constructed using the neighbor-joining method, and the numbers above the internal nodes indicate the percentages of 1000 bootstrap replicates that supported the branch.

#### *Anaplasma platys*

**Fig. 5:**
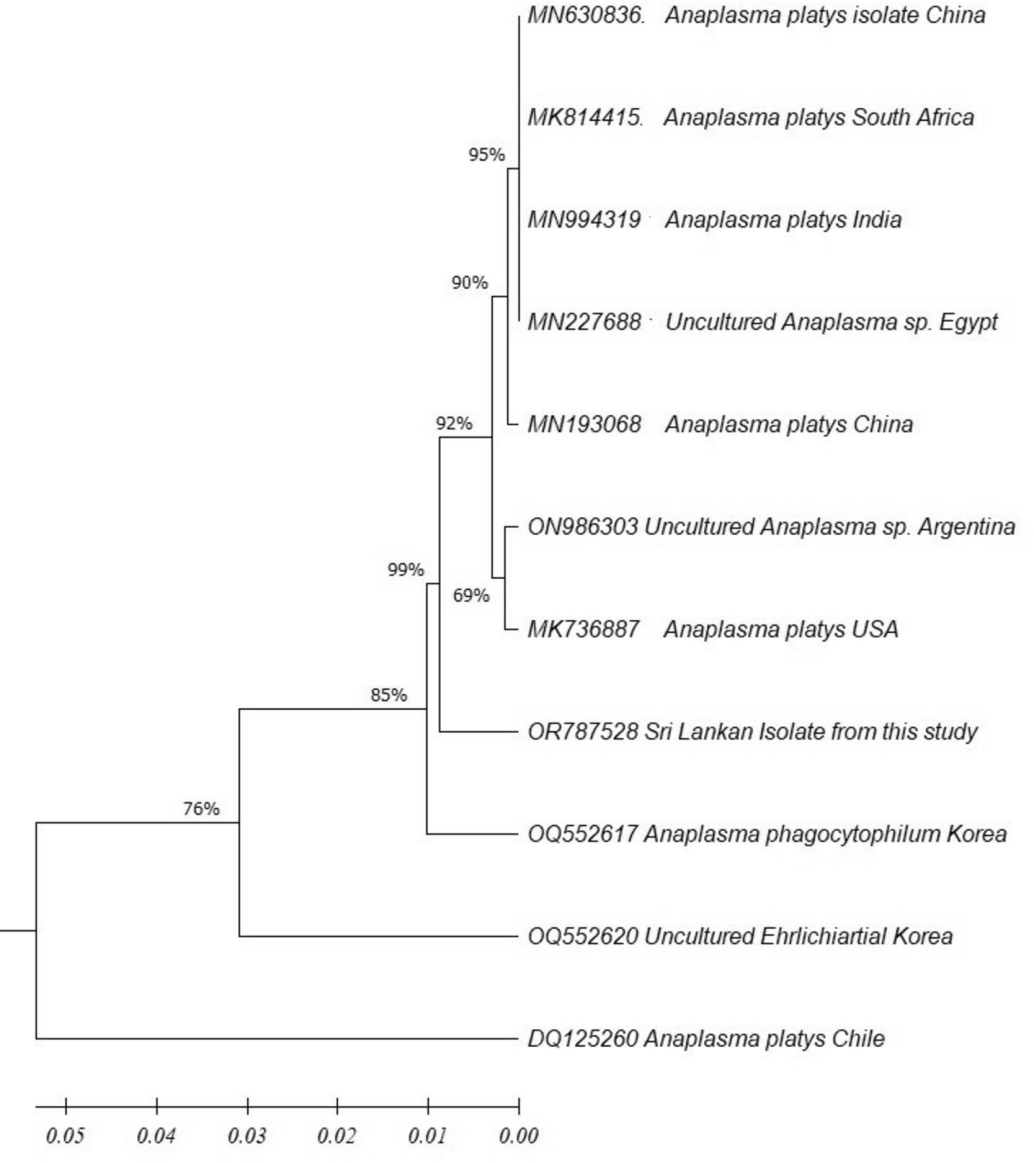
Phylogenetic tree of *Hepatozoon canis* based on the 18S ribosomal RNA (1*8S rRNA*) gene complete sequence with the neighbor-joining algorithm. (GenBank accession numbers are indicated after the species name)

The phylogenetic tree for *Anaplasma platys* DNA isolated 16S ribosomal rRNA partial sequence (GenBank accession number OQ44656) revealed the organism was closely related to *A. platys* isolates in USA (MK736887), Argentina (ON986303). China (MN 193068, MN630836), Egypt MN227688, India MN 994319 and South Africa MK 814415 and it is independent of *A. platys* reported in Chile (DQ125260) and Korea (OQ 552617, OQ552620) (Fig. 5). The tree was constructed using the neighbor-joining method, and the numbers above the internal nodes indicate the percentages of 1000 bootstrap replicates that supported the branch.

#### *Hepatozoon canis*

**Fig. 6:**
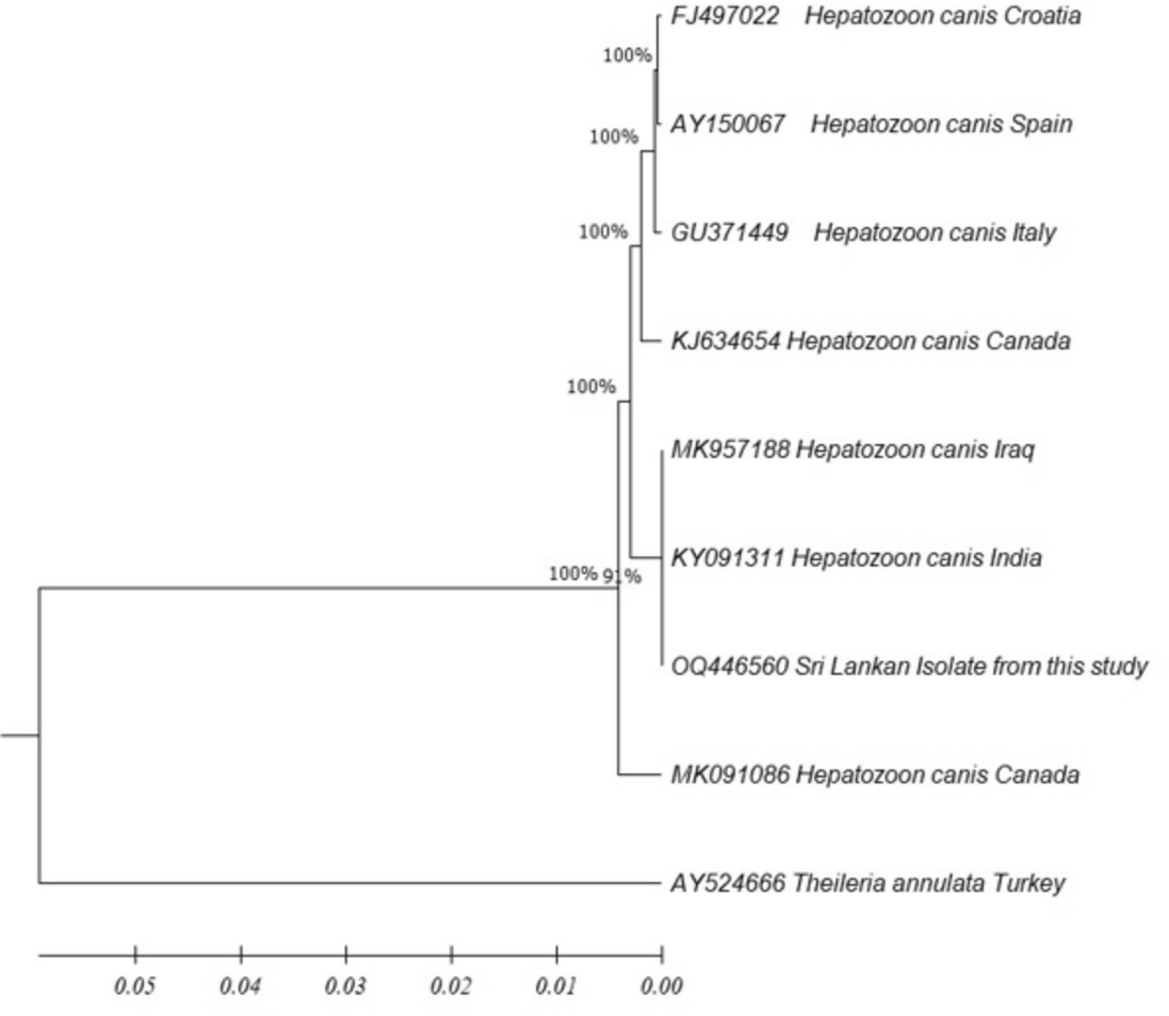
Phylogenetic tree of *Anaplasma platys* based on the 16 S ribosomal RNA (1*8S rRNA*) gene complete sequence with the neighbor-joining algorithm (GenBank accession numbers are indicated after species name)

The phylogenetic analysis of the sequence confirmed the species identified from this study as *Hepatozoon canis* (GenBank accession number OQ446560). It shows the close affinities to Indian KY091311 and Iraq MK957188 species (Fig. 6). The tree was constructed using the neighbor-joining method, and the numbers above the internal nodes indicate the percentages of 1000 bootstrap replicates that supported the branch.

#### *Leishmania* Sp.

**Fig. 7:**
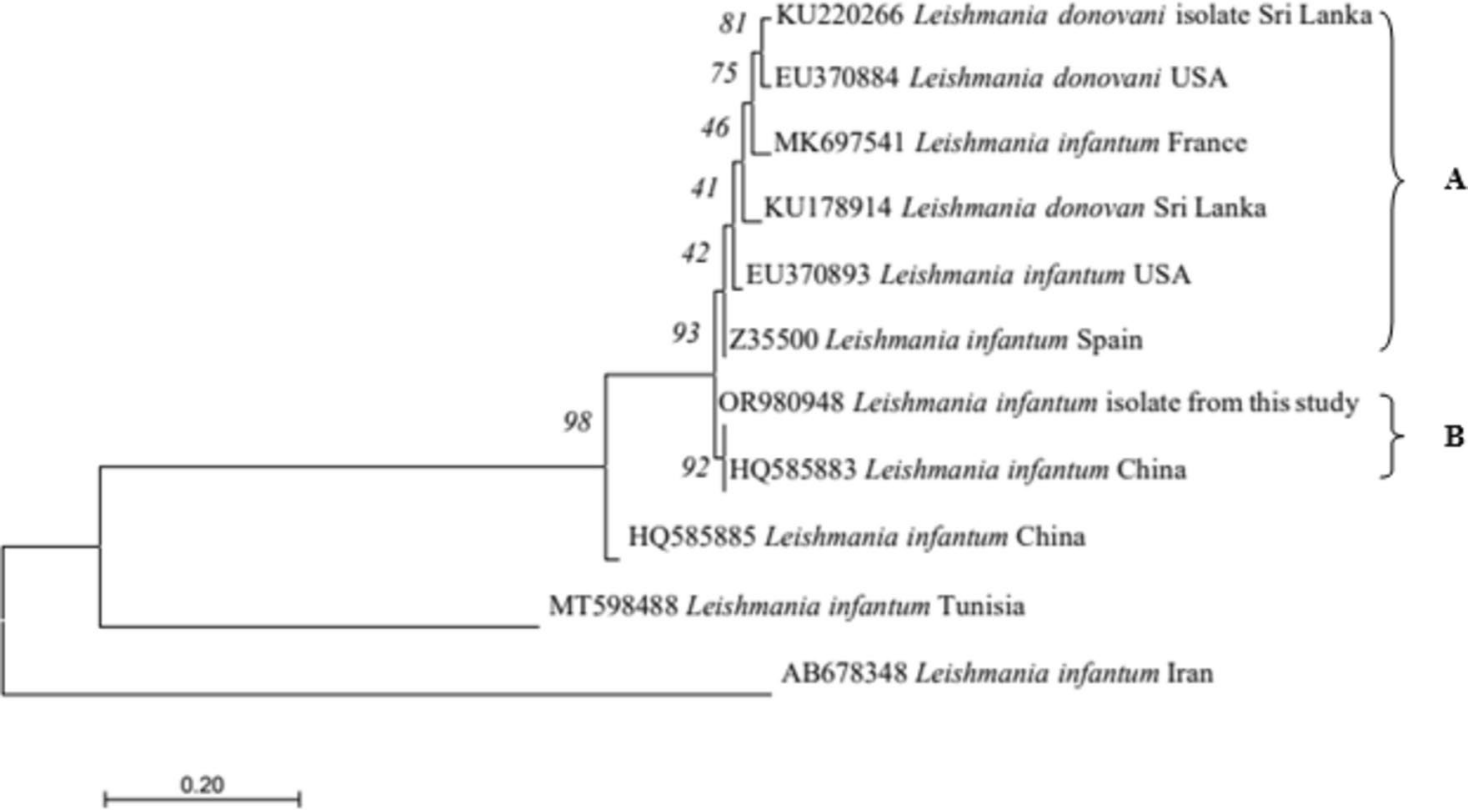
Phylogenetic tree of *Leishmania* based on kDNA gene partial sequence amplified using species-specific primers. The evolutionary history was inferred by using the Maximum Likelihood method based on the Tamura-Nei mode, 1993. The analysis involved one nucleotide sequence from isolates from the current study and previously published sequences in the GenBank. Numbers at the nodes represent percentage occurrences of clades based on 1000 bootstrap replications of data. Evolutionary analyses were conducted in Mega 7 (Kumar et al*.,* 2016)

Phylogenetic tree of *Leishmania* sp. based on kDNA gene partial sequence classified into two supported clusters (Fig. 7). The *Leishmania donovani* isolates identified previously in Sri Lanka from humans (KU220266, KU178914), as well as those from USA (EU370884) and, the *Leishmania infantum* isolates in USA (EU370893), France (MK697541) and Spain (Z35500) formed the first cluster (A). The *Leishmania* sp. isolated from this study (OR980948) formed the second cluster with *Leishmania infantum* isolates from China (HQ585883) showing close phylogenetic affinities.

### Geographic distribution of canine vector-borne infections

The number of dogs and the types of infections in the three SLAF establishments were different (Anuradhapura 8; Gampaha 161; Badulla 4). Still, there was no difference in disease prevalence among the dogs in the three SLAF locations (Fisher’s Exact Test, *p* > 0.05). Of the 18 districts, the Gampaha District had the highest prevalence (66.7%) followed by the Anuradhapura District (53.3%) and Ratnapura District had the lowest prevalence (10.0%) followed by Trincomalee (13.3%) of CVBIs among the FRDs and PODs (Table 3). The prevalence was within 20–40% in all the other districts.

**Table 3:** Distribution of pathogens in free-roaming and privately owned dogs in the 18 districts (blood smear positives).

District	Free–roaming							Owned dogs							
	n	Bg	Ec	Ap	L	F	Total	n	Bg	Bc	Ec	Ap	Hc	F	Total
Anuradhapura	5	2	1	–	–	–	3	10	3	–	1	1	–	–	5
Gampaha	10	3	1	–	2	1	7	5	2	–	–	–	–	1	3
Rathnapura	15	2	1	–	–	–	3	35	1	–	1	–	–	–	2
Galle	5	1	2	1	–	–	4	10	3	–	7	1	–	–	11
Colombo	25	3	2	1	–	–	6	40	4	1	2	–	1	–	8
Kaluthara	25	2	7	4	–	–	13	40	4	–	2	3	–	–	9
Matale	40	8	1	–	–	–	9	20	4	–	3	–	–	–	7
Vavuniya	10	1	–	–	–	–	1	5	1	–	1	–	–	–	2
Jaffna	20	1	2	1	–	–	4	20	5	–	4	1	–	–	10
Mulaitivu	5	–	1	1	–	–	2	15	2	–	3	1	–	–	6
Hambanthota	5	–	1	1	–	–	2	10	2	–	1	–	–	–	3
Kandy	5	1	–	–	–	–	1	10	4	–	–	–	–	–	4
Polonnaruwa	5	1	–	–	–	–	1	10	2	–	–	1	–	–	3
Trincomalee	5	2	–	–	–	–	2	10	–	–	–	–	–	–	0
Batticaloa	5	1	1	–	–	–	2	10	1	–	–	–	–	–	1
Puttlam	10	1	–	–	–	–	1	20	3	–	3	–	–	–	6
Kurunegala	5	–	1	–	–	–	1	10	2	–	1	–	–	–	3
Ampara	5	1	–	–	–	–	1	10	2	–	–	–	–	–	2
Total	205	30	21	9	2	1	63	290	45	1	29	8	1	1	85

District	Overall														
	n	Bg	Bc	Ap	Ec	Hc	L	F	Total						
Anuradhapura	15	5	–	1	2	–	–	–	8						
Gampaha	15	5	–	–	1	–	2	2	10						
Rathnapura	50	3	–	–	2	–	–	–	5						
Galle	15	4	–	2	9	–	–	–	15						
Colombo	65	7	1	1	4	1	–	–	14						
Kaluthara	65	6	–	7	9	–	–	–	22						
Matale	60	12	–	–	4	–	–	–	16						
Vavuniya	15	2	–	–	1	–	–	–	3						
Jaffna	40	6	–	2	6	–	–	–	14						
Mulaitivu	20	2	–	2	4	–	–	–	8						
Hambanthota	15	2	–	1	2	–	–	–	5						
Kandy	15	5	–	–	–	–	–	–	5						
Polonnaruwa	15	3	–	1	–	–	–	–	4						
Trincomalee	15	2	–	–	–	–	–	–	2						
Batticaloa	15	2	–	–	1	–	–	–	3						
Puttlam	30	4	–	–	3	–	–	–	7						
Kurunegala	15	2	–	–	2	–	–	–	4						
Ampara	15	3	–	–	–	–	–	–	3						
Total	495	75	1	17	50	1	2	2	148						

*Prevalence of infections in free-roaming and privately owned dogs; n = number of dogs sampled; Bg, *Babesia gibsoni*; Bc, *Babesia canis*; Ec, *Ehrlichia canis*; Ap, *Anaplasma platys*; F, Microfilaria; L, *Leishmania.*

### Distribution of infection among dog breeds

Breeds that were mostly infected in the SLAF were German Shepherd (36.1%), English Spaniel and Labrador Retrievers (25.1%), while among the PODs, Beagles (100%), Shih Tzu (100%), and Dachshund (83.3%) were infected (Table 4).

**Table 4:** Breed of the dogs and the haemoprarasites in the blood samples collected from the Sri Lanka Air Force military working dogs, free-roaming and privately owned dogs from 18 districts in Sri Lanka (Blood smear Positive).

	SLAF (%)			Free roaming (%)			Privately owned (%)			Overall (%)		
	Total	Single	Mixed	Total	Single	Mixed	Total	Single	Mixed	Total	Single	Mixed
Doberman (61)	2 (1.7)	1 (2.8)	1 (2.8)	–	–	–	4 (16.0)	4 (16.0)	0.0	6 (9.8)	5 (8.2)	1 (1.6)
German Shepherd (37)	13 (36.1)	12 (50.0)	1 (4.2)	–	–	–	4 (30.8)	3 (23.2)	1 (7.7)	17 (45.9)	15 (40.5)	2 (5.4)
Labrador Retriever (172)	23 (25.1)	21 (24.4)	1 (1.2)	–	–	–	20 (23.3)	19 (22.2)	1 (1.2)	42 (24.4)	40 (23.3)	2 (1.2)
Dachshund (6)	–	–	–	–	–	–	5 (83.3)	5 (83.3)	0.0	5 (83.3)	5 (83.3)	0
Beagle (7)	–	–	–	–	–	–	7 (100)	5 (71.4)	2 (28.6)	7 (100)	5 (71.4)	2 (28.6)
Pomeranian (12)	–	–	–	–	–	–	5 (41.7)	5 (41.7)	0.0	5 (41.7)	5 (41.7)	0
Rhodesian Ridgeback (28)	10	0.0	0.0	–	–	–	11 (61.1)	9 (50.0)	2 (11.1)	11 (39.3)	9 (32.1)	2 (7.1)
Terrier (5)	–	–	–	–	–	–	1 (20.0)	1 (20.0)	0.0	1 (20.0)	1 (20)	0
Boxer (6)	–	–	–	–	–	–	2 (33.3)	2 (33.3)	0.0	2 (33.3)	2 (33.3)	0
Shih tzu (3)	–	–	–	–	–	–	3 (100)	3 (100)	0.0	3 (100)	3 (100)	0
Cocker Spa(9)niel	–	–	–	–	–	–	3 (33.3	3 (33.3	0.0	3 (33.3)	3 (33.3)	0
Bullmastiff (4)	–	–	–	–	–	–	1 (25.0)	1 (25.0)	0.0	1 (25.0)	1 (25.0)	0
English Spaniel (7)	2 (28.6)	2 (28.6)	0.0	–	–	–	–	–	–	2 (28.6)	2 (28.6)	0
German Shepherd cross (2)	–	–	–	–	–	–	1 (50.0)	0.0	1 (50.0)	1 (50.0)	0	1 (50.0)
Doberman cross (2)	–	–	–	–	–	–	1 (50.0)	1 (50.0)	0.0	1 (50.0)	1 (50.0)	0
Boxer cross (1)	–	–	–	–	–	–	1 (100)	0.0	1 (100)	1 (100)	0	1 (100)
Rottweiler (13)	10	0.0	0.0	–	–	–	0.0	0.0	0.0	0.0	0	0
Mongrel (289)	–	–	–	205	43 (20.1)	11 (5.4)	7 (8.3)	5 (5.9)	2 (2.4)	61 (21.1)	48 (16.6)	13 (4.5)
Pug (4)	–	–	–	–	–	–	0.0	0.0	0.0	0.0	0	0
Total	39 (22.5)	36 (20.8)	3 (1.7)	44 (21.5)	43 (20.9)	11 (5.4)	76 (26.2)	66 (22.8)	10 (3.5)	668	145 (21.7)	24 (3.6)

### Clinical signs and asymptomatic cases

Pathogens were found both in dogs with clinical signs and without clinical signs. Anorexia, lethargy, fever, pale mucosa, dark urine, epistaxis, skin bleeding on the ventral abdomen, and emaciation were the most common clinical signs. Besides, dogs showed the clinical signs mentioned above, but the blood samples were microscopically and DNA negative for infections (SLAF dogs 23.3% and PODs 11.3%). The percentages of infected dogs with clinical signs and without clinical signs were comparable in SLAF (without signs 41.1% with signs 58.9%) and PODs (without signs 38.2% with signs 61.8%), having more individuals with clinical signs. However, among the infected FRDs, a large number (96.3%) did not show signs while only 3.7% showed signs and this was significantly higher compared to SLAF and PODs (Chi-square test, *p* < 0.0001) but there was no difference between SLAF dogs and PODs (χ^2^ = 0.0050, *p* > 0.938). All the dogs at the SLAF with mixed infections showed clinical signs, while none of the FRDs with mixed infections showed any signs. The SLAF dogs with no clinical signs rarely had parasites compared to other categories and if they do show, it is primarily due to *B. gibsoni* followed by *E. canis* and *A. platys*. Among the breeds, Labrador Retrievers mostly showed symptoms, including anorexia, lethargy, fever, pale mucosa, dark urine, epistaxis, skin bleeding on the ventral abdomen, and emaciation. Among the free-roaming dogs, only two from Katunayake were clinically ill, showing signs of skin disease. Both these dogs were infected with *Leishmania* sp. This parasite was not found in any other dog in the entire sample.

### Comparison of morphological and molecular diagnosis

Out of the seven parasites, diagnosis of *B. gibsoni* using only microscopy was not sensitive as 17 dogs were smear-negative but were DNA positive for *Babesia.* Out of these, 16 were FRDS and one was a POD. Microcopy results showed 15.6% were smear-positive, while molecular analysis showed 23.4% were DNA-positive.

Ticks were collected from 30 FRDs (n = 85), 20 PODs (n = 47) and 10 SLAF dogs (n-55). Three tick species were identified: *Rhipicephalus linneai* (formerly known as *Rhipicephalus sanguineus* sensu lato tropical lineage), *Rhipicephalus haemaphysaloides* and *Haemophysalis bispinosa.* Among them, SLAF and PODs had only *R. linneai*, while FRDs had *R. haemaphysaloides* and *H. bispinosa* in addition to *R. linneai*. Both nymphs and adults of *R. linneai* and only the adult stages of *R. haemaphysaloides* and *H. bispinosa* were reported.

## Discussion

This study reports the first comprehensive and comparative, island-wide investigation of CVBIs among the PODs, FRDs and military working dogs of the SLAF. Even though all the parasite species have been previously recorded in Sri Lanka, those studies are confined to one or a few sites or focused on a particular parasite species. The CVBIs of military working dogs in SLAF have not been studied before. Overall, more than one-fourth of the dogs examined were infected, mostly as single infections and a few mixed infections. Infections in FRDs were significantly higher than in the PODs or SLAF dogs. A similar study carried out in Tamil Nadu, India testing *Anaplasma, Babesia, Ehrlichia, Hepatozoon*, filarioids and *Leishmania* reported a very high overall prevalence of 67.8% (n = 156) examining 230 dogs^33^. Another study with 2,104 dogs comprising a stray dog population in Assam, India and a hospital population including privately owned pet dogs and working dogs of the Central Parliamentary Forces reported 57.31% infected comprising 58.03% in pets, 54.54% in the working dogs and 63.64% in stray dogs^34^. A study from Eastern Austria, tested 94 clinically healthy military working dogs for the presence of filaria worms, *Leishmania,* piroplasms, *Borrelia, Bartonella* and Anaplasmatacease and showed that two dogs were positive for *D. repens* and six clinically healthy dogs (4.2%) were positive for *Babesia canis* and 10.6% were seropositive for *Borrelia burgdoferi* s.l.^29^. There is no difference in infection levels among the three dog categories in their study^29^, and the percentages of infected dogs are higher than those reported in the present study. Among the infected dogs, FRDs were often more asymptomatic than the other two dog categories. However, there was no difference between the SLAF and PODs. This is anticipated since better natural resistance against CVBIs in stray dogs than PODs or pure breeds of dogs is well established^35–37^. The absence of clinical signs indicates that these dogs may be chronically or sub-clinically infected with these pathogens, or as Dantas-Torres and Otranto^38^ pointed out, they may be having clinical pathological abnormalities. Although chronic infections may not pose an immediate threat to the animals, these dogs do remain possible reservoirs for infections, stressful conditions, concurrent illnesses, pregnancy, and parturition, which may precipitate clinical signs in chronically infected animals^39,40^.

The FRDs are sub-clinically infected and may provide a continuous source of infection for these pedigree dogs. Arthropod vectors can transmit these infections from FRDs to PODs, mostly to pedigree dogs which are selectively bred to conform to the aesthetic value of the dog rather than its health and, therefore, frequently suffer from the effects of inbreeding as the gene pool available is highly limited. Studies have shown that such breeding practices could have increased the expression of inherited defects and thus compromised the health and welfare of many breeds^41–44^. The reduced heterozygosity of a highly inbred population can contribute to the frequency of occurrence of inherited disease in the population^43,45^. The top 50 most popular breeds of pedigree dogs in the UK are predisposed to 312 inherited disorders, with German shepherd dogs and Golden retrievers associated with the most significant number of ailments^46^. Many CVBIs in healthy hunting dogs from Central Italy and confirmed that dogs infected by these pathogens often develop asymptomatic or subclinical forms^47^. Another study in Turkey^48^ reported a lower percentage of 5.4% out of 757 asymptomatic domestic dogs infected with vector-borne rickettsia and protozoans. The presence of CVBIs in asymptomatic dogs is relevant from an epidemiological point of view, as the transmission potential of symptomatic and asymptomatic dogs can vary depending on the parasite species. For example, some studies have shown that asymptomatic dogs cannot infect vectors with *Leishmania*^49,50^. Others demonstrate that transmission occurs in a similar proportion as that for oligosymptomatic animals, but to a lesser extent than for symptomatic dogs^51–56^. It is important to investigate whether these asymptomatic dogs serve as reservoirs.

The present study reported seven infections: *Babesia gibsoni, Babesia canis, Ehrlichia canis, Hepatozoon canis, Leishmania* sp.*, Anaplasma platys* and *filaria worms*. Out of these, *B. gibsoni, E. canis* and *A. platys* were recorded in all three dog categories. Among these, *B. gibsoni* was the most prevalent pathogen island-wide (13.8%). There was no difference in the prevalence of *B. gibsoni* among the three dog categories. Only one case of *B. canis* was reported from a POD. Even though *B. gibsoni* was the most prevalent canine small morphotype, for large form, three main species of *Babesia* exist in dogs: *B. canis, B. vogeli, and B. rossi*^57^. The primary vectors for these pathogens differ: *Dermacentor reticulatus, Haemaphysalis elliptica*, and *R. sanguineus* for *B. canis, B. rossi* and *B. vogeli* respectively^57^. In Tamil Nadu India, *R. sanguineus* sensu lato is the major tick vector and *R. haemaphysaloides* to a lesser extent (less than 2%)^33^. The major dog tick is *R. linneai* (formerly *R. Sanguineus* sensu lato) and *Rh. haemaphysalodes* in Sri Lanka^58^; however, their vector capacity has not been studied. Globally, babesiosis is a common vector-borne disease among domestic and wild canines^59^.

A study from the Anuradhapura  District in Sri Lanka reported *B. gibsoni* and *B. canis* (with a prevalence of 15.0% and 1.3%, respectively) in addition to mixed infections in three Divisional Secretariat Divisions: Rambewa, Tirappane, and Galenbidunuwewa^6^. A recent study investigated canine babesiosis in dogs brought to the Veterinary Teaching Hospital at the University of Peradeniya and showed a high prevalence of *B. gibsoni* (78.6%) in the Kandy District^7^. In Tamil Nadu India, two species of *Babesia* are reported and *Babesia vogeli* (10%), as the more prevalent species than *B. gibsoni* (0.4%)^33^. However, the study conducted on the stray dog population in Assam, India, reported *B. gibsoni* as the most prevalent infection, with 47.16% in hospital dogs and 47.72% in stray dogs^34^. In the present study, all the FRD (100%) smear-positive for babesiosis (31 dogs) were asymptomatic, while 46.7% of the SLAF. dogs and 27.5% of the PODs were asymptomatic. Asymptomatic babesiosis has been reported elsewhere with a prevalence of 3.42% (29 of 848) cases of asymptomatic dogs in Croatia^60^. The prevalence of babesiosis could be higher in these dogs as Ranatunga et al.^7^ reported that 33.3% of blood smear-negative dogs were PCR-positive for *Babesia* DNA.

*Ehrlichia canis* infection was the second most prevalent canine hemoparasite, with a similar prevalence as *B. gibsoni* infection (15 dogs). None of the smear-positive, FRDs (12 dogs) showed any signs, while 62.5% of SLAF dogs and 50% of PODs were asymptomatic. Infection with *E. canis* may result in acute disease, chronic disease or remain clinically silent^8^. Moreover, due to the non-specific and variable symptoms, often misdiagnosed or diagnosed past the point of recovery which can be fatal^61^. A higher prevalence of *E. canis* infections (56.1%)^6^ compared to the previously reported prevalence of 14% in dogs in the Western Province^62^. Although in Tamil Nadu in India, *E. canis* infection was as high as 16.1%^33^, in Assam, India, the military dogs infected with *E. canis* were much less (< 3%), comparatively^34^. *Ehrlichia canis* has a worldwide distribution, and dogs and other canids are the natural hosts. It is generally not considered a zoonotic agent, but some cases of human infection have been reported in Venezuela^63^.

*Anaplasma platys* infection was also reported in all three dog categories, with a higher prevalence among the SLAF dogs (6.4%) compared to other dog categories. The first record of *A. platys* (formerly known as *Ehrlichia canis*) in Sri Lanka was in 2005 from Colombo using buffy coat analysis and confirmation by PCR^8^ reported 18% owned dogs and 12% stray infected while 75% with no clinical signs. The study carried out in the Anuradhpura District didn't report *A. platys*^6^. *Anaplasma platys* is also more common in Tamil Nadu, India (22.6%)^33^ and among the working dogs in Asam, India (8.49%)^34^. In addition, *Anaplasma phagocytophilum* was also reported at a low prevalence (0.4%) in Tamil Nadu^33^. Anaplasmosis is an emerging infectious disease affecting dogs in many parts of the world and can be manifested as acute or non-clinical infections^64^.

*Leishmania* was found only in two FRDs as a single infection with a prevalence of 0.9%. Two previous studies reported a very low infection rate. In 151 dogs, only two *Leishmania* amastigotes were recorded in Giemsa-stained smears (prevalence 1.3%), one in the skin and one in peripheral blood^16^. Another study examined 114 stray dogs and only one (0.9%) showed detectable anti-*Leishmania* sp. antibodies^15^. Since serological assays frequently lack specificity, it is hard to distinguish between species and frequently cross-react with *Trypanosoma* and other trypanosomatids^65^. These studies show that the prevalence of canine leishmaniasis may not be a widespread CVBI but further studies are needed to confirm its occurrence as its zoonotic potential has been highlighted^16^. Human leishmaniasis is an emerging infection caused by *Leishmania donovani* which is traditionally considered a visceralizing anthroponotic species but causes cutaneous leishmaniasis in Sri Lanka^66^. In the present study, both infected dogs showed clinical signs. Asymptomatic dogs, some even without skin parasitism, are competent in transmitting *Leishmania* to the sandfly vector^67^. However, some^38^ argue that the term "asymptomatic" is of limited value because it does not consider clinical-pathological abnormalities and those with organ dysfunction^68^ and recommend the LeishVet guidelines of^69^ for those who are involved in research in canine Leishmaniasis. In India, the presence of *Leishmania* has been attributed to domestication of dogs by tribes^70^. Canine leishmaniosis due to *L. infantum* is enzootic in some countries, and it is an emerging zoonosis in endemic foci. Although phylogeny showed close affinities to *L. infantum*, further studies are needed for confirmation.

Only one POD was infected with *H. canis*. Acute hepatozoonosis in five dogs has been characterized by neurological symptoms, ataxia orparesis, emaciation and anaemia^10^. Recently, in Galenbindunuwewa in the Anuradhapura District in Sri Lanka, *H. canis* has been reported as a single infection (1.56%) and as mixed infection with *B. gibsoni* and *B. canis* (1.56%)^6^. *Hepatozoon canis* is a common CVBI reported from several parts of India and in Tamil Nadu as the most prevalent infection among dogs in (37.8%)^33^ and other parts of India^71^ and is distributed throughout the Old World. Disease associated with the infection is usually asymptomatic, while disease, when present, may range from subclinical and chronic, especially in the absence of concurrent infections, to severe and life-threatening^72^.

Filaria worms were also found in all three dog categories but were always as mixed infections either with *B. gibsoni* in FRDs and PODs or with *B. canis* and *A. platys* in the SLAF dogs. Canine filariasis has been reported previously from Sri Lanka and the species identified include *D. repens*, *B. ceylonensis* and *B. malayi,* and their geographic distribution and prevalence varied from 30 to 68.8%^11–13, 73, 74^. Mallawarachchi et al.^11^ anticipate that the actual rates of infections are even higher. However, the prevalence of filaria worms in the present study was 0.6% with only four dogs being infected. All the canine filaria worms recorded in Sri Lanka are zoonotic [see^11^] and can cause disease in humans. In 2016, Sri Lanka received the WHO certification to eliminate lymphatic filariasis or bancroftian filariasis^75^; however, the emergence of zoonotic canine filariasis may endanger the filariasis-free status of the country due to the potential reservoirs for humans.

Between the two methods of diagnosis 23.4% were DNA-positive while only 15.6% were smear-positive. A study carried out in Kerala, south India, 71 (47.33%) were found to be PCR positive for *B. gibsoni*, while only 40 were blood smear positive^76^ and similar findings have been reported in other studies^77,78^. Liu et al.^79^ report a QubeMDx PCR system that enables a rapid, sensitive and reliable diagnosis of *B. gibsoni* near the dog patient. Within 30 min, this diagnostic assay can detect parasitemia as low as 0.002% in the dog blood, providing a reliable point-of-care test to assist in diagnosing *B. gibsoni*.

The pattern of infection was similar in the island-wide SLAF dogs and the FRDs. However, it varied in the PODs. Socio-economic factors of the dog owners and their capability or willingness to afford to use methods to control ectoparasites contribute to the level of infection among PODs^79^. The SLAF veterinarians claim a thorough quarantine and screening process for all the imported dogs, even to detect infections at subclinical levels before introducing them to the military units. They have likely acquired the diseases through the tick vectors once they are brought to the country. Nymphs and adults of *R. linneai* were found on the SLAF dogs while the FRDs had *R. haemaphysaloides* and *H. bispinosa* in addition to *R. linneai.* Among the ticks infesting dogs, *Rh. linneai* is the dominant species in the Dry and Wet zones, while *R. haemaphysaloides* was the dominant in the intermediate zone of Sri Lanka while *H. bispinosa is* also a common tick in dogs^80^.

The FRDs may act as reservoirs of these diseases as a substantially high population of stray dogs is found island-wide. As a strategy to suppress the spread of rabies, the Rabies Ordinance of 1893 allowed FRDs to be seized and disposed of. However, in 2006, a presidential order was passed to implement a “no-kill policy,” and with the lobbying of animal activists, a more humane approach of the “catch-neuter-vaccinate-release” method (CNVR) was practiced. The statistics show a dramatic decline in reported rabies cases, but these FRDs act as constant reservoirs of CVBIs by habouring the vectors of these infections.

Environmental changes affect emerging parasitic diseases^81^. As Dantas-Torres reviewed in 2015, human developments affect the environment and the climate, affecting biodiversity and altering tick population dynamics and CTBI transmission^2^. The present study provides baseline data on the types of infections and prevalence of these diseases in the SLAF kennels, FRDs, and PODs in Sri Lanka. They can be used in future studies of disease dynamics, vectors of infections, and seasonality, together with additional knowledge on ticks and other vectors, animals, pathogens, and their interactions with the whole ecosystem.

## Methods

Ethical clearance for the study protocols was obtained from the Ethical Review Committee at the Postgraduate Institute of Science, University of Peradeniya, Sri Lanka. All experimental protocols were approved, and the methods were carried out in accordance with the ARRIVE guidelines.

### Study animals

Although no census has been carried out, the dog population in Sri Lanka is estimated at around 2.5 million^31^. These dogs can be categorised as (1) privately owned and (2) free-roaming stray dogs. Privately owned dogs usually stay inside the house or enclosed garden and are taken out only for defaecation/urination and daily exercise. They are routinely vaccinated, dewormed, and regularly taken to a veterinary hospital or clinic. Free-roaming stray dogs that do not have an owner and feed on garbage sometimes hunt other animals and are not vaccinated or given any veterinary care unless during acute sickness or severe injury. They may be vaccinated through mobile clinics but not dewormed. Free-roaming strays are shy of people with a higher possibility of close contact with wild animals. Samples were collected from adults (more than one-year-old) and puppies (less than one-year-old).

Sri Lanka has dogs belonging to many breeds. Some of the privately owned dogs are purebred; some are crossbred with other breeds or with the local Sinhala hound or Sinhalese hound belonging to the species Sinhala “sunakaya”, which is found in Sri Lanka and parts of India^32^ (they are called mongrels from here onwards). The SLAF dogs are mostly imported, and a few are bred locally. The SLAF also has its dog breeding station at Diyathalawa. These SLAF dogs are used in various tasks, including explosive detection, tracking, guard dogs, lifesaving, dog shows, security, demining, and narcotic detection.

### Study sites, clinical data, ectoparasites and blood sample collection

The required minimum sample size was calculated using the Creative Research Systems survey software (http://www.surveysystem.com/sscalc.htm)*,* considering a 50% expected hemoparasite prevalence, with an acceptable 10% variation at 95% confidence interval level. Blood samples were collected from the cephalic vein of the dogs at the three SLAF establishments (Katunayake, Anuradhapura and Diyathalawa; Fig. 1). Samples were also collected from healthy dogs close to these SLAF establishments and distributed island-wide (both free-roaming and privately owned) brought to the government and private veterinary care facilities in the district with the support from the field veterinarians. The blood samples were transferred into 3 mL ethylenediaminetetraacetic acid (EDTA) tubes for smears and PCR analysis and were stored at 4 °C until processed. Information on whether the dogs had clinical signs suggestive of infections was also collected for individual dogs from the respective handler, owner, or a known party for stray dogs. Samples were taken from the following categories of dogs: (a) SLAF with clinical signs, (b) SLAF without clinical signs, (c) free-roaming dogs living in proximity to the SLAF, (d) privately owned dogs brought to the veterinary clinic with clinical signs, (e) privately owned dogs brought to the clinic without clinical signs. Ectoparasites of dogs were collected whenever possible, preserved in 95% ethanol and identified using available keys and literature^58,82^. Sample collection was carried out from July 2016 to July 2019.

### Giemsa-stained thin blood smears

Thin blood smears were prepared from all the dogs, air dried, stained with Giemsa (10%), and examined under the light microscope on oil immersion (at 1000× magnification) for pathogen identification using keys^83,84^.

### DNA extraction and PCR amplification

All the samples were subjected to molecular identification using appropriate primers. DNA was extracted from 200 µL of whole blood using ReliaPrep™ Blood gDNA Miniprep System (Promega, Madison, USA). The extracted DNA was subjected to PCR to check the presence of *Babesia*, *Leishmania*, *Anaplasma*, *Hepatazoons*, and *Ehrlichia*. The PCR amplified the 18S rDNA gene of *Babesia* and *Hepatozoon* and 16S rDNA gene of *Ehrlichia* and *Anaplasma* and kDNA gene of *Leishmania*. The Primer details and PCR programmes for each primer pair are given in Table 5. Each PCR reaction mixture (30 µL) included 15 µL of Go Taq master mix, 0.5 µL of each forward and reverse primer, 3 µL of template DNA, and adequate nuclease-free water. The amplified PCR products were separated using ethidium bromide-stained agarose gel (1%) electrophoresis and visualized under UV illumination.

**Table 5:** Details of PCR primers used in this study and reaction conditions.

Target parasite	Primer	Oligonucleotide sequences	Fragment size (bp)	PCR protocol	References
*Babesia* (*18S rRNA*)	B18SF B18SR	F: GTTTCTGMCCCATCAGCTTGAC R: CAAGACAAAAGTCTGCTTGAAAC	1463–1647	95 °C for 5 min initial denaturation, followed by 35 cycles of 95 °C for 1 min, 55 °C for 1 min, 72 °C for 1.30 min, then 72 °C for 10 min for the final extension	85
*Ehrlichia* *Anaplasma* (*16S rRNA*)	GE2f. EHRL3-IP2	F: GTTAGTGGCATACGGGTGAAT R: TCATCTAATAGCGATAAATC	136–152 bp	95 °C for 5 min initial denaturation, followed by 35 cycles of 95 °C for 1 min, 50 °C for 1 min, 72 °C for 1.30 min, then 72 °C for 10 min for the final extension	86
*Hepatozoon* (*18S rRNA*)	Hep F Hep R	F: ATACATGAGCAAAATCTCAAC R: CTTATTATTCCATGCTGCAG	666 bp	The amplification protocol was employed in a thermal cycler (2720, Applied Biosystems, Foster City, CA, USA) as following: 9 5 °C for 12 min (for polymerase activation), followed by 34 cycles of 95 °C for 30 s (denaturation); 57 °C for 30 s (annealing); 72 °C for 1 min and 30 s (extension), followed by 7 min at 72 °C (final extension)	87
*Leishmania* (*kDNA*)	Leish 150 Leish 152	F: GGG(G/T)AGGGGCGTTCT(C/G)CGAA R:(C/G)(C/G)(C/G)(A/T)CTAT(A/T)TTACACCAACCCC	120 bp	PCR amplifications were performed in a 96-well Verit Thermal Cycler (Applied Biosystems) using the following program: initial denaturation at 96 °C for 6 min, followed by 40 cycles of 30 s at 93 °C, 30 s at 64 °C, and 30 s at 72 °C, with a final extension at 72 °C for 7 min. Five microliters of the amplification reaction product were resolved on a 1.5% agarose gel and visualized under UV transillumination	88

### DNA sequencing and phylogeny

PCR-amplified products of *Babesia, Ehirlichia, Anaplasma, Hepatazoon* and *Leishmania* (18S rRNA, 16S rRNA and kDNA) were sequenced for species identification and phylogenetic analysis. The PCR products were purified using the QIAquick PCR purification kit (Qiagen, Hilden, Germany), and sequencing was conducted using the Genetic analyzer 3500 series (Applied Bio Systems^®^). The resulting sequences of each isolate were compared for new sequences to other published sequences available in GenBank using NCBI-BLAST (http://www.ncbi.nlm.nih.gov /BLAST). Unique sequences were deposited in the GenBank database. The nucleotide sequences were aligned using ClustalW (24) with the previously published sequences in the GenBank for phylogenetic analysis. Aligned sequences were trimmed to the same length (with gaps), from which phylogenetic trees were constructed based on the neighbor-joining (NJ) tree method using the program MEGA7 (version 7) with suitable models.

### Data analysis

Results were presented as proportions and percentages in tables, while a chi-square test or a Fisher’s Exact test (when expected frequencies were less than 5) at 5% significance was performed at appropriate degrees of freedom when required to compare among and between categories.

## Data Availability

All data are available in hard copies and soft copies with the principal investigator stored securely releasable upon any reasonable request.
